# Human and animal cystic echinococcosis in Tataouine governorate: hypoendemic area in a hyperendemic country, myth or reality?

**DOI:** 10.1186/s13071-021-04714-5

**Published:** 2021-04-21

**Authors:** Selim M’rad, Raja Chaâbane-Banaoues, Massaouda Ghrab, Hamouda Babba, Myriam Oudni-M’rad

**Affiliations:** 1 Laboratory of Medical and Molecular Parasitology–Mycology (LP3M), LR12ES08, Faculty of Pharmacy, University of Monastir, 5000 Monastir, Tunisia; 2 Laboratory of Parasitology–Mycology, EPS F. Bourguiba, 5000 Monastir, Tunisia

**Keywords:** Cystic echinococcosis, *Echinococcus granulosus*, Epidemiology, Serodiagnosis, Prevalence, Genotyping, Environmental contamination, Humans, Livestock, Dog faeces

## Abstract

**Background:**

Cystic echinococcosis (CE) has a worldwide distribution and is especially prevalent in North African countries. With a mean annual surgical incidence (ASI) of CE of 12.7 per 100,000 inhabitants, Tunisia is one of the most CE endemic countries in the Mediterranean area. Tataouine governorate is considered to be the most CE hypoendemic region in Tunisia (ASI = 0.92) despite favourable socioeconomic conditions that enable maintenance of the *Echinococcus granulosus* *sensu lato* (*s.l*.) life-cycle and a significant environmental contamination with *E. granulosus* *s.l*. eggs. The aim of this study was to assess human CE seroprevalence, prevalence of CE in food animals and environmental contamination by *E. granulosus s.l.* eggs in different districts of Tataouine governorate.

**Methods:**

This study was conducted from January to December 2018. A total of 374 human sera samples were tested for the presence of immunoglobulin G antibodies against *E. granulosus* using a commercial ELISA kit. Specimens were also collected from animals slaughtered at the Tataouine abattoir (*n* = 8609) and examined for the presence of hydatid cysts; 111 hydatid cysts were genotyped. Eggs of *E. granulosus* *s.l.* were identified by PCR and DNA sequencing from dog faecal samples (*n* = 288).

**Results:**

Serological tests showed that 8.5% of the sera samples tested were positive for *E. granulosus*-specific antibodies. The average prevalence of hydatidosis in livestock was 1.6%, and CE infection was more prevalent in cattle than in sheep, goats and dromedaries. The contamination rate of dog faeces by *E. granulosus sensu stricto* eggs varied significantly from 0 to 23.5% depending on the collection area. Molecular analyses only revealed the presence of the G1 genotype for cysts and eggs.

**Conclusions:**

Based on our findings, CE is likely to be more endemic in the Tataouine governorate than previously described. Thus, to implement an effective control programme against CE, a national survey should be carried out to determine human CE prevalence in the different Tunisian governorates.

**Graphic Abstract:**

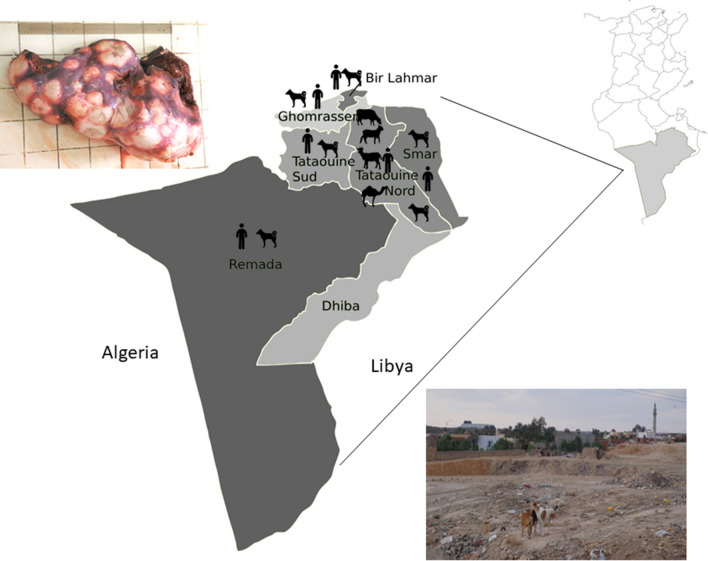

## Background

Cystic echinococcosis (CE), also known as hydatidosis, is a zoonotic disease caused by the larval stage of the species complex *Echinococcus granulosus sensu lato* (*s.l*.). CE has a worldwide distribution and particularly affects poor and pastoral rural communities. Human CE constitutes a severe food-borne parasitic disease listed among the neglected diseases prioritised by the World Health Organization [1]. The annual economic loss due to this disease is estimated at around USD 3 billion worldwide [2] and USD 10–19 million in Tunisia [3]. The life-cycle of the parasite requires two hosts: canids as definitive hosts and a wide range of herbivorous or omnivorous animals as intermediate hosts. The adult tapeworm inhabits the small intestine of canids, especially dogs, which release eggs in their faeces. After ingestion by a grazing intermediate host, the viable oncosphere develops into a larval form, or hydatid cyst, mainly in the liver and the lungs. Human infection is due to accidental ingestion of eggs through contaminated vegetables, water, soil and fomites or by direct contact with infected dogs [4]. The liver and the lungs are the most commonly involved organs although many anatomic sites may be affected [5].

*Echinococcus granulosus s. l.* is a complex composed of at least five cryptic species: *E. granulosus sensu stricto* (*s.s*) (G1 and G3 genotypes), *E. equinus* (G4 genotype), *E. ortleppi* (G5 genotype), *E. canadensis* (G6–G10 genotypes) and *E. felidis* [6–9]. *Echinococcus granulosus* *s.l.* has a worldwide distribution, but is especially prevalent in North African countries [10, 11]. In Tunisia, *E. granulosus* *s.s.* (particularly the G1 genotype) is the most common species associated with human and animal CE [12, 13].

Human diagnosis relies essentially on imaging techniques (ultrasonography, conventional radiography, computerized axial tomography and/or magnetic resonance imaging), and serological tests, such as the enzyme-linked immunosorbent assay (ELISA), indirect haemagglutination assay and immunoblot assay [14]. The classical treatment of human hydatid cyst is surgery, but anthelmintics (albendazole, 10 mg/kg per day) can be administered in pre- and post-surgical settings [5].

With a mean annual surgical incidence (ASI) of CE of 12.7 per 100,000 inhabitants, Tunisia is one of the most endemic countries in the Mediterranean area [15]. CE is a notifiable disease in Tunisia, and based on the findings of the latest national survey on CE, the 24 governorates (administrative units) were classified as: hyperendemic (ASI > 19), holoendemic (12.7 < ASI < 19), mesoendemic (6.3 < ASI < 12.7) and hypoendemic regions (ASI < 6.3) [16]. The governorate of Tataouine is currently considered to be the most hypoendemic area for CE in Tunisia (ASI = 0.92). However, two studies conducted on dog faecal samples collected in Tataouine governorate highlighted a high environmental contamination with *E. granulosus* *s.s.* eggs [17, 18]. Thus, it seems relevant to clarify the hypoendemic status of this governorate in such a hyperendemic country as Tunisia. The aim of this epidemiological study was to assess CE human and animal prevalence and environmental contamination by *E. granulosus* *s.s.* eggs in Tataouine governorate.

## Methods

### Study area

**Fig. 1:**
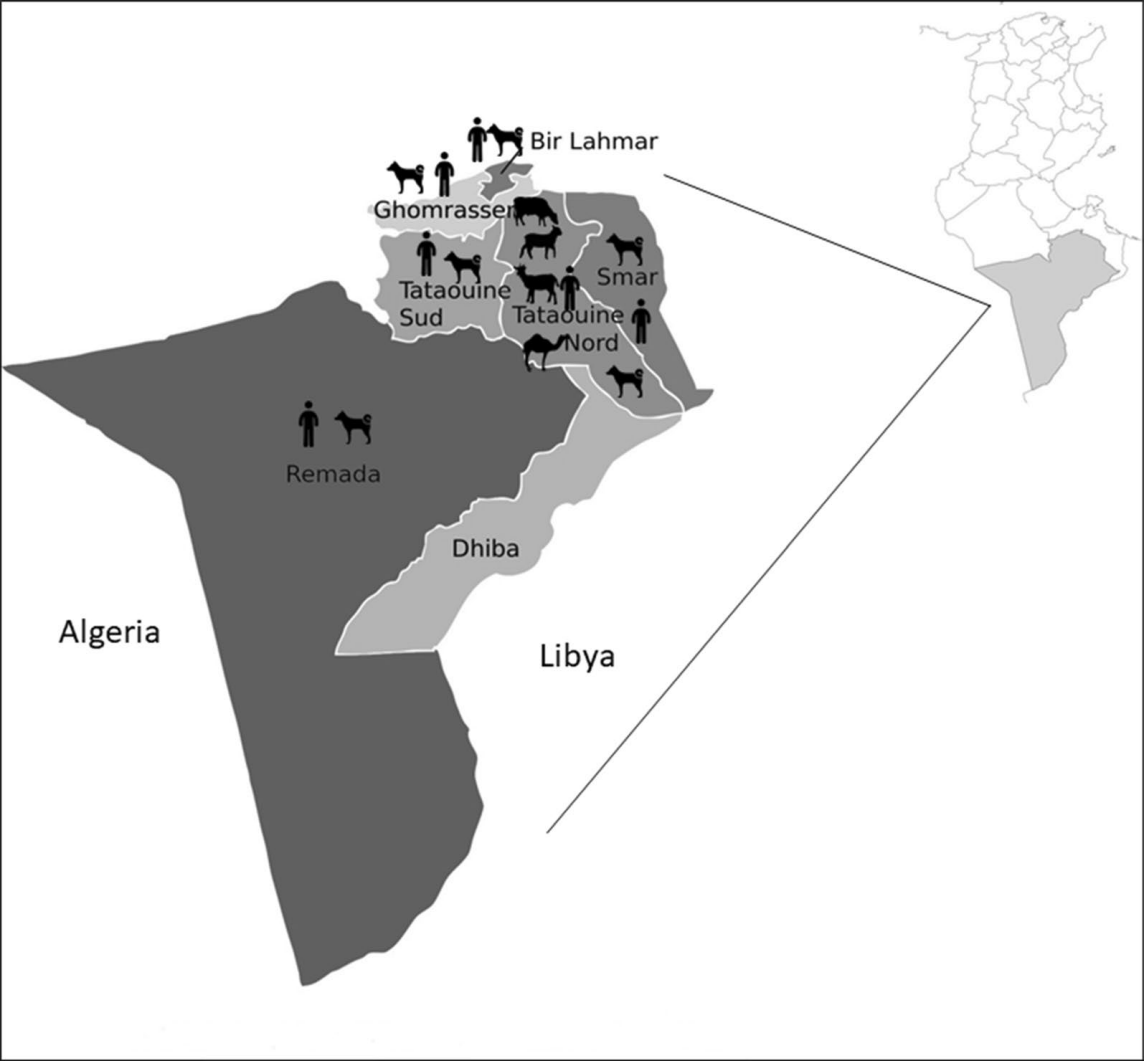
Locations (districts) in Tataouine governorate where the samples (human sera, dog faeces and livestock cysts) used in this study were collected

The study was conducted in the governorate of Tataouine, southeastern Tunisia (32°55′40″N, 10 26′57″E), located 531 km from the capital city of Tunisia (Tunis) (Fig. 1).

Covering an area of about 40,000 km^2^ (20% of the total Tunisian area), Tataouine is the largest governorate in Tunisia, with a population of 150,830 inhabitants [19]. The governorate is divided into seven districts (Bir Lahmar, Ghomrassen, Tataouine Nord, Tataouine Sud, Remada, Smâr and Dhiba). It is characterised by a desert climate, with an annual average temperature of 22 °C, summer time temperatures exceeding 40 °C and an annual rainfall ranging between 88 and 157 mm. Agricultural activities are carried out on 17,000 km^2^ of Tataouine governorate, with grazing by 5.5 million heads of sheep and goats (10% of the total population) and 25,000 heads of camelids (50% of the total population) [20].

### Sample collection

#### Human samples

The study was carried out in two stages. In the first stage, we conducted a retrospective study of the medical records of patients who visited the Regional Hospital of Tataouine for consultation for a CE serodiagnosis during a 12-year period (2000–2012). Patients’ personal data, such as age, gender and district of origin, were registered. In the second stage, we performed an investigator blind test of CE serological diagnosis on a pre-existing collection of human serum samples collected in 2018. All patients included in this investigator blind test were from different districts of Tataouine governorate (Fig. 1). A total of 374 sera samples were collected from these patients at Tataouine Hospital for blood tests (full blood count, erythrocyte sedimentation rate, fasting glucose level) requested for reasons unrelated to CE. For all of these patients, gender and district of origin were listed; age was only recorded for 320 patients (Table 1). The patients providing the samples ranged in aged from 1 to 85 years. A serological test for CE diagnosis was performed using a commercial ELISA kit (Ridascreen® Echinococcus IgG ELISA; R-Biopharm, Pfungstad, Germany) following the manufacturer’s instructions. Optical density (OD) was measured in a photometer at 450 nm using a reference wavelength ≥ 620 nm. OD results were used to calculate and interpret a sample index (SI). ELISA results were considered to be positive at SI ≥ 1.1, negative at SI < 0.9 and equivocal at 0.9 ≤ SI < 1.1. The borderline results were considered to be negative.

**Table 1:** Characteristics of patients seropositive for immunoglobulin G antibodies against *Echinococcus granulosus* investigated by enzyme-linked immunosorbent assay for cystic echinococcosis in terms of sex, age and district of origin

Variables	Groups	Seroprevalence		*P*-value^a^
		Number of examinated patients (%)	Number of positive patients (%)	
Sex	Female	275 (73.5)	23 (8.4)	0.484^b^
	Male	99 (26.5)	9 (9.1)	
	Total	374 (100.0)	32 (8.6)	
Age (years)	0–9	23 (7.2)	2 (8.7)	0.782
	10–19	34 (10.6)	4 (11.8)	
	20–29	54 (16.9)	4 (7.4)	
	30–39	56 (17.5)	5 (8.9)	
	40–49	41 (12.8)	1 (2.4)	
	50–59	50 (15.6)	7 (14.0)	
	60–69	29 (9.1)	2 (6.9)	
	70–79	21 (6.6)	1 (4.8)	
	80–89	12 (3.8)	4 (33.3)	
	Total	320 (100.0)	30 (9.4)	
District	Tataouine Nord	190 (50.8)	19 (10.0)	0.176
	Tataouine Sud	142 (38.0)	11 (7.7)	
	Smâr	4 (1.1)	0 (0)	
	Remada	28 (7.5)	1 (3.6)	
	Bir Lahmar	3 (0.8)	0 (0)	
	Ghomrassen	7 (1.9)	1 (14.3)	
	Total	374 (100.0)	32 (8.6)	

^a^*P*-value according to Chi-square/Fisher’s exact test, as indicated

^b^Fisher’s exact test

#### Faecal samples

A total of 284 dog faecal samples were collected between January and December 2018 from six different rural and semi-urban districts (Bir Lahmar, Ghomrassen, Tataouine Nord, Tataouine Sud, Remada and Smâr) of Tataouine governorate (Fig. 1). The different collection areas are all in the vicinity of towns, located about 20–40 km apart. The faecal samples of stray and unrestrained dogs were collected from the ground, conserved in a clean plastic container without alcohol or formalin fixation and frozen at − 80 °C for 7 days to inactivate the infective eggs. For all faecal samples, Taeniidae eggs were concentrated using the sucrose flotation technique as described by Chaâbane-Banaoues et al. [17] and then identified by light microscopic examination [21]. Recovered Taeniidae eggs were indistinguishable from one another by microscopic observation, thus *E. granulosus* *s.s.* egg identification was performed by Egss1 PCR assay and cytochrome* c* oxydase subunit 1 (*Cox 1*) sequencing. Briefly, DNA was extracted using a phenol/choroform protocol subsequent to an alkaline lysis (dithiothreitol and potassium hydroxide) and an enzymatic digestion by proteinase K (Invitrogen, Carlsbad, CA, USA) for 1 h at 56 °C [17]. The mitochondrial 12S rRNA gene was amplified with PCR using the Egss1 primer set specific to the G1 genotype of *E. granulosus* *s.s.* [22]. The PCR assay was carried out as previously described by Hizem et al. [23]. The non-amplified Egss1 fragments were re-examined targeting a fragment of the mitochondrial *Cox1* gene by PCR assay according to the procedure described by Gasser et al. [24]. *Cox1* PCR products were sequenced and aligned with BLASTn program (https://blast.ncbi.nlm.nih.gov/Blast.cgi).

#### Livestock samples

A total of 8609 animals (6622 goats, 1854 sheep, 11 cattle and 122 dromedaries), slaughtered between January and December 2018 at the central abattoir located at Tataouine Nord district (Fig. 1), were examined for the presence of *E. granulosus* hydatid cyst. A total of 111 hydatid cysts (67 from goats, 30 from sheep, 13 from cattle and 1 from a dromedary) were collected during meat inspection using visual examination, palpation and systematic incision of the visceral organs. Calcified or degenerated cysts were not considered in the present study. Cyst fertility (presence or absence of protoscoleces) was determined by light microscopic observation. The protoscolex viability was assessed using a vital 0.2% eosin staining. For 80 cysts (45 from goats, 24 from sheep, 10 for cattle, and 1 from a dromedary), DNA was directly extracted from the protoscoleces if present, using the phenol/chloroform protocol [25], or from the germinal layer using a Chelex®-100 chelating resin method (Bio-Rad, Hercules, CA, USA). Thus, an isolate refers to the protoscoleces or the germinal layer obtained from a single hydatid cyst. The presence of the G1 genotype of *E. granulosus* *s.s.* was confirmed using the Egss1 PCR as described in [23].

### Statistical analysis

Qualitative variables were summarized as counts and percentages, and differences were analysed with Fisher’s exact test, Chi-square test and two-proportion Z-test, as appropriate, with significance values set at  *P* < 0.05. The 95% confidence interval (95% CI) was calculated. Correlation between human serological prevalence (percentage of patients seropositive for CE) and environmental infestation indexes (percentage of faecal samples positive for *E. granulosus* eggs) was assessed using Pearson’s correlation. All data analyses were carried out using SPSS® statistical software, version 20 (IBM Corp., Armonk, NY, USA).

## Results

### Cystic echinococcosis in humans

A thorough study of the clinical records of patients consulting for CE over a 12-year period revealed that only four serological CE diagnoses were requested by the treating doctors. Of these patients, three were seropositive for immunoglobulin G (IgG) antibodies against *E. granulosus*, a result confirmed by computerized tomography. Two patients were men aged 19 and 78 years who presented with hepatic cysts, and the third was a 47-year-old woman with a pelvic cyst. They were treated with albendazole, 10 mg/kg per day, before surgery.

The mean age of the population studied in the investigator blind test was 40 ± 20.82 years. A marked predominance of females was noted (73.5* vs* 26.5%). The district of origin, age, and gender distribution of patients with CE are summarized in Table 1.

Serological tests indicated that 32 of the 374 sera samples tested (8.6%; 95% CI: 5.7–11.36) were positive for *E. granulosus*-specific IgG antibodies. The mean age of these seropositive patients was 42 ± 24.18 years (Table 1). No significant differences in hydatidosis occurrence between females (8.4%) and males (9.1%) (*P* = 0.484) and between the age groups (*χ*^2^ = 2.463, *df* = 8, *P* = 0.782) were recorded (Table 1). Seropositivity ranged from 0 to 14.3% depending on the district, with no statistically significant between-district (*χ*^2^ = 10.595, *df* = 5, *P* = 0.176; Table 1).

### Faecal sample contamination

Among the 288 dog faecal samples tested, 37 (12.8%) were contaminated with taeniid eggs (Table 2). *Toxocara* spp. (16 isolates) and *Trichuris* spp. (2 isolates) eggs and protozoan oocysts (5 samples) were also observed (data not shown).

**Table 2:** Distribution of dog faeces contaminated with *E. granulosus* *s.s.* eggs according to district

District	Ghomrassen	Tataouine Sud	Tataouine Nord	Remada	Smâr	Bir Lahmar	Total	*P*-value^a^
Total number of faecal samples examined	80	33	84	28	34	29	288	
Positive for taeniid eggs, *n* (%)	10 (12.5)	3 (9.1)	16 (19)	0 (0)	8 (23.5)	0 (0)	37 (12.8)	0.009
Positive for *E. granulosus* (*s.s.*) eggs, *n* (%)	8 (10)	2 (6)	14 (16.6)	0 (0)	8 (23.5)	0 (0)	32 (11.1)	0.007

^a^*P*-value (according to Chi-square test)

The G1 genotype of *E. granulosus* *s.s.* was identified for 32 taeniid egg samples (11.1%; 95% CI: 7.7–15.3). For the five remaining isolates, the sequencing products showed 99–100% homology to *T. hydatigena* for three faecal isolates (GB accession number #KT372520.1) and to *T. multiceps* for two isolates (GB #LC271734.1) when they were compared against the NCBI database.

The contamination of dog faeces with *E. granulosus* *s.s.* eggs varied significantly from 0 to 23.5% depending on the district (*χ*^2^ = 16.011, *df* = 5, *P* = 0.007) (Table 2). The isolates from Remada and Bir Lahmar districts were free of *E. granulosus* *s.s.* eggs. Pearson’s correlation test revealed no relationship between faecal sample contamination and human seropositivity in the studied districts (*r* = − 0.181, *P* = 0.720).

### Cystic echinococcosis in livestock

During the post-mortem examination, 8609 domestic ruminants were inspected for hydatid disease. Among these, 113 goats, 24 sheep, three cattle and one dromedary harboured one or more cysts with a diameter ranging from 1.5 to 15 cm (Table 3). The average CE prevalence was 1.6% (95% CI: 1.4–1.9). The CE prevalence differed significantly between animal species and was more extensive in cattle (27.3%; 95% CI: 6.0–60.9) than in goats (1.7%; 95% CI: 1.4–2.0), sheep (1.3%; 95% CI: 0.8–1.9) and dromedaries (0.8%; 95% CI: 0.2–4.4) (*χ*^*2*^ = 43.45, *df* = 3, *P* < 0.001) (Table 3).

**Table 3:** Prevalence of cystic echinococcosis in slaughtered animals during the study period

Species	*N*	Infected animals, *n* (%)	*P-*value^a^
Goat	6622	113 (1.3)	*P* < 0.001
Sheep	1854	24 (1.7)	
Cattle	11	3 (27.3)	
Dromedary	122	1 (0.8)	
Total	8609	141 (1.6)	

*N*, Number of examined animals; *n*, number of infected animals

^a^*P*-value (according to Chi-square test)

A greater number of cysts were collected from the liver (56.7%) than from the lungs (43.2%) (*Z* = 1.879, *P* = 0.024) (Table 4). The mean fertility rate was low (29.7%) but the cysts from the lungs were more fertile than those from the liver (50* vs* 14.3%, *Z* = − 3.868, *P* < 0.001) (Table 4). For fertile cysts, the protoscolex viability was estimated to be 80% (data not shown). The molecular genotyping of the 80 collected cysts (60 infertile and 20 fertile cysts)revealed the presence of only the G1 genotype of *E. granulosus* *s.s.*

**Table 4:** Organ localization and fertility assessment of collected cysts from animals slaughtered in Tataouine governorate during the study period

Organ localization and fertility assessment	Total (*N* = 111)	Goat (*n* = 67)	Sheep (*n* = 30)	Cattle (*n* = 13)	Dromedary (*n* = 1)	*P-*value^a^
Infection according to localization						
Liver	63 (56.7)	43 (64)	18 (60)	1 (8)	1 (100)	0.024
Lung	48 (43.2)	24 (36)	12 (40)	12 (92)	0 (0)	
Fertility rate						
Fertile	33 (29.7)	16 (23.9)	13 (43.3)	3 (23.1)	1 (100)	< 0.0001
Infertile	78 (70.3)	51 (76.1)	17 (56.7)	10 (76.9)	0 (0)	
Fertility according to localization						
Liver	9 (27.3)	3 (18.75)	5 (38.5)	0 (0)	1(100)	< 0.0001
Lung	24 (72.7)	13 (81.25)	8 (61.5)	3 (100)	0 (0)	

Values in the tables are presented as a number with the percentage in parentheses

^a^*P*-value (according to Z-test)

## Discussion

Despite current preventive control measures (i.e. destruction of contaminated offal and euthanasia of roaming dogs), CE remains a public health concern in Tunisia where the CE endemic status varies from one region to another. In the present study, the serological diagnosis revealed that human CE seroprevalence in Tataouine governorate was significant (8.6%). This high seroprevalence was unexpected considering the fact that the ASI in Tataouine governorate is the lowest in Tunisia [15] and that only three CE seropositive patients were recorded in our retrospective study over a 12-year period. Moreover, the prevalence reported in the present study was higher than that reported in the neighbouring country of Libya (2.3%) [26]. Tunisia is a country with a low socioeconomic status, especially in the southern region where it is not always easy to have access to medical follow-up and good quality healthcare. A major proportion of the population of Tataouine governorate lives in rural areas with poor health/hygienic facilities [27]. There is a severe lack of medical facilities in this region; consequently, serological diagnosis of CE and surgery on patients with CE are frequently performed in hospitals in other governorates, which may explain the low serological diagnosis reported here.

No significant relationship between age and CE seroprevalence was noted, although CE is often described as a young adult infection that commonly starts during childhood. Considering that human exposure to *E. granulosus* *s.l.* eggs continues throughout life, it is expected that adults might be more exposed than children and have a higher probability of ingesting viable *E. granulosus* *s.l.* eggs [4]. Therefore, the difference observed in the present results compared to the national ASI data might be explained by the fact that hydatid cysts grow slowly and may remain asymptomatic for years or decades before triggering clinical symptoms. Moreover, the ASI reported for the Tataouine governorate is outdated and human CE prevalence has very likely changed in the intervening years. Indeed, in the context of the Tunisian post-revolutionary period and the international economic crisis, poverty has risen, hygienic conditions have deteriorated and slaughterhouse control has decreased, all factors facilitating the establishment of the life-cycle of the parasite.

Several studies conducted in Tunisia, Algeria and Libya have reported that women are more commonly affected than men due to the female role in domestic activities, especially in rural areas, and their frequent contact with dogs, soil and vegetables [23, 26, 28, 29]. The absence of significant differences in CE prevalence between men and women in the Tataouine governorate might be attributed to the differences in lifestyle in this region where men are more implicated in agricultural practices and it is rare for women to be in contact with dogs.

Human seroprevalence was not different on the basis of district of origin, and no relationship was observed between human CE seropositivity and contaminated dog faecal samples, as previously been demonstrated in a large-scale study conducted in seven governorates [17]. The highest percentage of dog faecal samples contaminated with *E. granulosus* *s.s.* eggs was found in Smâr; however, CE human seropositivity was one of the lowest at this location. The overall contamination of dog faeces by *E. granulosus* *s.s.* eggs (11.1%) was lower than that reported for the whole country (25.3%) but similar to that previously described for Tataouine governorate (14.6%) [17]. These differences in the contamination rate of dog faeces might be due to different canine density and/or total number of tapeworms per dog. In Tunisia, one of the main factors associated with the disease is the density of dogs per square kilometer and per inhabitant, followed by levels of illiteracy in the population, the proportion of households not connected to sewage networks and the proportion of the population in rural areas [16]. Although Chaâbane et al. [18] demonstrated that environmental contamination by *E. granulosus* *s.s.* eggs is not related to the presence of slaughterhouses, this is probably not the case in this study where Tataouine Nord, the district containing the largest slaughterhouse in the region, presented one of the highest faecal contamination rates. The numerous *E. granulosus* *s.s.* eggs in the environment of Tataouine are partly due to poorly and inadequately equipped slaughterhouses, limited economic resources and the absence of efficient waste disposal facilities, as is the case in several North African countries [10, 30, 31]. In this context, and despite the efforts of health authorities, the destruction of contaminated offal is not always implemented, making them easily accessible to free roaming dogs.

On the other hand, home (during cultural and religious celebrations) and uncontrolled (in little roadside restaurants) slaughtering is frequent in Tunisia. Thus, inappropriate human behaviour regarding the management of infected offal and people’s unawareness of the risks associated with feeding dogs with such raw viscera also contribute to the maintenance of the life-cycle of *E. granulosus* *s.s.* [16, 31, 32]. In Tunisia, the canine population, estimated at about 800,000 dogs, is essentially composed of stray and unrestrained dogs that are often fed with raw discarded offal [33]. In the northern and central regions of the country, the prevalence of *E. granulosus* *s.s.* in dogs varies between 5 and 21%, with the total number of tapeworms per dog ranging from 2 to 2540 [34, 35], resulting in a massive dissemination of *E. granulosus* *s.s.* eggs into the environment. At a larger scale, studies performed in neighbouring countries, such as Algeria and Libya, demonstrated a similar prevalence of *E. granulosus* *s.l.* in necropsied dogs of 16 and 16.1% (range: 6.7–22.5%), respectively [36, 37]. Unfortunately, no investigation into the prevalence in dogs was carried out in the Tataouine governorate. Nevertheless, even though the prevalence of *E. granulosus* *s.s.* in dogs is lower than that in other governorates, the eggs remain viable over months to years and remain a risk for public health.

The CE prevalence in slaughtered livestock recorded here is low (1.6%) and similar to that reported in 2017 (1.7%) in the Tataouine governorate by the Regional Commissary for Agricultural Development of the Tataouine Governorate. This prevalence varied considerably depending on the animal species. Except for cattle, the prevalence was lower than that reported in the other governorates of Tunisia where *E. granulosus* *s.s.* infection ranged from 16.42 to 40.42% in sheep and was 8.5% in cattle, 6% in dromedaries and 2.9% in goats [38, 39]. Tunisia is a country with a past and current history of pastoral husbandry, especially in the southern regions where traditional livestock breeding systems are based on the use of large rangelands [20]. The differences in prevalence observed in the present study might have been influenced by the level of environmental contamination by *E. granulosus* *s.s.* eggs and the climatic conditions (low rainfall and high temperature in summer) that reduce the viability of eggs [40], but also by livestock age at slaughter. It is known that the *E. granulosus* *s.l.* contamination is higher in older than in younger animals because older animals have been more exposed to *E. granulosus* *s.l.* eggs throughout their life [39]. It is possible that livestock in Tataouine governorate are slaughtered at a younger age compared other governorates, but no data on the age of the slaughtered animals were available for the present study.

Even if CE animal prevalence is relatively low, this does not prevent the maintenance of the life-cycle. A recent survey was conducted in India over a 7-year period to determine the prevalence of CE in slaughtered animals, in dogs and in humans [41]. The study demonstrated that despite low infestation rates ranging from 0.01 to 3.00% in livestock and no more than 4.34% in dog faeces, a high CE human seroprevalence (11%) was present.

Contrary to other governorates where sheep is the most consumed animal [39], in Tataouine governorate the goat is the most commonly slaughtered animal. As reported in several studies, goats seem to be less frequently infected than sheep, cattle and dromedaries, and a weak CE infection of goats was described in the northern governorates of Tunisia (2.9%, *n* = 3779) [39], Algeria (0%, *n* = 37) [42] and Morocco (1.88%, *n* = 2337) [43]. This lower infection rate might result from goats’ feeding behaviour which consists mostly of grazing on bushes and tree branches, making them less exposed to the ingestion of *E. granulosus* *s.s.* eggs.

In Tunisia, the G1 genotype of *E. granulosus* *s.s.* remains the prevailing genotype involved in animal CE, and only some studies have reported genotype G6 in camel infections [12, 13, 44]. This finding is similar to that reported in Maghreb countries, such as Algeria, Libya and Morocco, where CE transmission essentially implies a domestic life-cycle involving dogs and livestock (reviewed in Deplazes [11]). The predominance of the G1 genotype in Tunisia could be related to its probable role in its dissemination in neighbouring and distant countries. The authors of a recent study carried out on the near-complete mitochondrial genome sequences argue that Tunisia might be one of ancestral locations of the G1 genotype and possibly be responsible for several diffusion routes of the G1 *E. granulosus* *s.s.* to Algeria and Morocco* via* the dispersal of domesticated animals, and to Argentina, Australia and Turkey* via* human and livestock migration [45].

The main limitation of this study is that diagnosis was restricted to a blind investigator ELISA test in the absence of imagery to confirm results. Due to the retrospective nature of the work, data retrieval was very challenging, and there was no system in place that enabled us to contact CE seropositive patients and to confirm our serological results by imagery. Moreover, the ELISA commercial kit that was used can detect not only IgG antibodies against *E. granulosus* but also those against *E. multilocularis*, which raises the possibility of potential false positive results. Nevertheless, since only two human alveolar echinococcosis cases have been reported over the last 35 years in Tunisia, it is quite unlikely to have had an impact on our results [46, 47]. The manufacturer has also reported cross-reactions with *Taenia solium* antigens. Nonetheless, with Tunisia being a Muslim country, it is unlikely that *T. solium*, which needs pigs as intermediate hosts, can complete its life-cycle in Tunisia.

## Conclusions

The findings of this study demonstrate that human CE infection and environmental contamination by *E. granulosus* *s.s.* eggs are high in a governorate considered to be hypoendemic for CE. The Tataouine governorate is probably more endemic for CE than previously described, and there seems to be an existing socioeconomic situation that is favourable for cystic echinococcosis transmission in this governorate. There are numerous social factors favouring the maintenance of the *E. granulosus* *s.s.* life-cycle, such as poor public awareness of CE, home slaughter of livestock, improper and uncontrolled disposal of infected viscera and frequent exposure of stray dogs by *E. granulosus* *s.s.* contaminated offal.

To better understand the CE epidemiological situation in the Tataouine governorate, especially for humans, further investigations, including prospective serological studies combined with medical imagery, are needed. To implement an effective control programme against CE, a national survey conducted on humans, livestock and dogs should be carried out in order to better assess the CE endemic status of the different Tunisian governorates.

## Data Availability

The datasets used and/or analyzed during the current study are available from the corresponding author on reasonable request.

## Abbreviations

ASI: Mean annual surgical incidence
CE: Cystic echinococcosis
95% CI: 95% Confidence intervals
*Cox 1*: Cytochrome *c* oxydase subunit 1
ELISA: Enzyme-linked immunosorbent assay
OD: Optical density
SI: Sample index
